# *Gpr174* Knockout Alleviates DSS-Induced Colitis *via* Regulating the Immune Function of Dendritic Cells

**DOI:** 10.3389/fimmu.2022.841254

**Published:** 2022-05-20

**Authors:** Wei Wei, Sucheng Mu, Yi Han, Yao Chen, Zhongshu Kuang, Xingyue Wu, Yue Luo, Chaoyang Tong, Yiqun Zhang, Yilin Yang, Zhenju Song

**Affiliations:** ^1^Department of Emergency Medicine, Zhongshan Hospital, Fudan University, Shanghai, China; ^2^Endoscopy Center and Endoscopy Research Institute, Zhongshan Hospital, Fudan University, Shanghai, China; ^3^ Shanghai Key Laboratory of Lung Inflammation and Injury, Shanghai, China; ^4^Shanghai Institute of Infectious Disease and Biosecurity, School of Public Health, Fudan University, Shanghai, China

**Keywords:** GPR174, inflammatory bowel disease, dendritic cells, intestinal barrier function, T cell activation

## Abstract

**Background:**

Dysfunction of the immune system would disturb the intestinal homeostasis and lead to inflammatory bowel disease (IBD). Dendritic cells (DCs) help maintain intestinal homeostasis and immediately respond to pathogens or injuries once the mucosa barriers are destroyed during IBD. G protein-coupled receptors(GPR)174 is an essential regulator of immunity that is widely expressed in most immune cells, including DCs. However, the role of GPR174 in regulating the immune function of DC in colitis has not been investigated.

**Methods:**

Dextran sodium sulfate (DSS) was administered to establish the mice colitis model. Data of weight, length of colon, disease activity index (DAI), and macroscopic scores were collected. The flow cytometry was used to detect the infiltrations of T cells and DCs, the mean fluorescence intensity (MFI) of CD80, CD86, CD40, and major histocompatibility complex-II (MHC-II). And T cells proliferataion was measured by carboxyfluorescein diacetate succinimidyl ester (CFSE). The expression of cytokines (tumor necrosis factor-α (TNF-α), interleukin-6 (IL-6), interleukin-10 (IL-10), interferon-γ (IFN-γ), interleukin -4 (IL-4)) and *GPR174* mRNA were measured by Elisa, quantitative polymerase chain reaction (qPCR), and immunofluorescence. RNA of bone-marrow-derived dendritic cells (BMDCs) was extracted for sequencing. Adoptive transfer of BMDCs was administrated intravenously.

**Results:**

*Gpr174*^-/-^ mice exposed to 3% DSS showed significant alleviation characterized by reduced loss of weight, more minor colon damage, and better DAI and macroscopic scores. The expression of pro-inflammatory cytokines (TNF-α, IL-6) decreased, while anti-inflammatory cytokine (IL-10) increased compared with WT mice. *In vitro*, *Gpr174^-/-^* BMDCs showed less maturity, with a declined expression of MHC-II, CD80, CD86 and reduced TNF-α, higher IL-10 after LPS stimulation. *Gpr174^-/-^* BMDCs were less capable of activating OT-II naïve CD4^+^ T cells than WT BMDCs and induced more Th0 cells to differentiate into Treg while less into Th1. Furthermore, the transcriptome sequencing analysis exhibited that *Gpr174* participated in TNF-α (NF-κB) signaling, leukocyte transendothelial migration, and Th1/Th2 cell differentiation pathways. Adoptive transfer of *Gpr174^-/-^* BMDCs to WT mice ameliorated DSS-induced colitis.

**Conclusion:**

Our study indicated that *GPR174* was involved in the pathogenesis of IBD by regulating the maturation of the dendritic cells to maintain immune homeostasis. TNF-α (NF-κB) signaling pathway, leukocyte transendothelial migration, and Th1/Th2 cell differentiation pathways may be the target pathway.

## Introduction

Inflammatory bowel disease (IBD), consisting of Crohn’s disease (CD) and ulcerative colitis (UC), is a multifaceted disease resulting from genetic, autoimmune, and environmental factors, which in fact, is an inflammatory condition of the gastrointestinal tract (1). Although precise pathogenesis is not clear, the immune response caused by the invasion of intestinal pathogens has been regarded as a pathogenic factor. The sustaining and aberrant inflammation responses disturb the mucosa and mucosal immunity homeostasis, leading to inappropriate and magnified intestinal inflammation (2).

Dendritic cells (DCs) maintain intestinal homeostasis and respond at the first time to pathogens or injuries once the mucosa barriers are destroyed during IBD (3). Clinical evidence indicates that the numbers and subsets of CD103^+^ dendritic cells change in inflamed mucosa, display loss of tolerogenic function, and disorder of cytokine profiles in IBD patients (4, 5). Similarly, dextran sulfate sodium salt (DSS)-induced colitis is developed in severe combined immunodeficiency (SCID) mice, suggesting that acute DSS colitis did not require the presence of either T cells or B cells, while dendritic cells participate in the pathogenesis of IBD independently (6, 7).

G protein-coupled receptors (GPCRs) are critical signaling molecules in immune response, cell proliferation, inflammation regulation, and intestinal barrier maintenance (8). One of the GPCRs, GPR174, is widely expressed in the intestine, spleen, thymus, and lymph nodes (9), is widely expressed in most immune cells, including T/B lymphocytes, and DCs, and is involved in many infectious diseases, including sepsis (10, 11). *Gpr174* has also been reported to play an important role in gender dimorphism of humoral immunity and be more susceptible to experimental autoimmune encephalomyelitis. It suppresses germinal center formation in male mice and is a chemokine receptor for the CCL21 ligand (12).

However, whether GPR174 could regulate the immune function of DCs in IBD has not been investigated. The present study aimed to identify GPR174 involved in colitis by both *in vivo* and vitro studies. In addition, we focused on the relationship between GPR174 and DCs function, which may play a critical role in regulating the intestinal injury of colitis.

## Methods

### Animals

Male wild-type C57BL/6 and male *Gpr174* knockout mice (*Gpr174*^-/-^) (8-12 weeks, 20-25g) were obtained from the Southern Model Biological Technology Development Co. (Shanghai, China) and bred under pathogen-free conditions. Animals were housed separately in a temperature-controlled room with a 12-hour light/12-hour dark cycle and free food and water access. All experimental procedures and animal operations followed the international guidelines for the Care and Use of Laboratory Animals (NO. 201804001Z).

### Induction and Assessment of DSS-Induced Colitis

The acute colitis mice model (n=25) was induced by oral administration of 3% DSS (35-50 kDa)(Sigma-Aldrich, St. Louis, MO, United States) for seven days and two days on regular drinking water (13). The control group of mice (n=25) was only fed with regular drinking water during the study. The health condition was monitored daily, and the disease activity index (DAI), including weight loss, stool consistency, and stool bleeding, was scored as described (14). All mice were sacrificed on day 9 after the intraperitoneal injection of avertin, and the colon was collected for length and gross macroscopic appearance (15).

### Intestinal Permeability Assay

Intestinal permeability was measured by fluorescein isothiocyanate (FITC)-dextran (3,000-5,000 kDa) (Sigma-Aldrich, St. Louis, MO, United States) (16). Briefly, 9 days after oral administration of DSS, mice were fasted for 4 h and then gavaged with FITC-dextran (0.5 mg/g body weight at 125 mg/mL). Four hours later, blood taken from the abdominal aorta was centrifuged at 12,000 g for 5 min. The serum was collected to detect the FITC-dextran by a microplate reader with an excitation wavelength of 490 nm and an emission wavelength of 520 nm.

### Myeloperoxidase (MPO) Assay

The MPO activity assay was applied to assess the neutrophil infiltration into the colon. The distal colon was homogenized in 4 volumes of MPO Assay buffer (Jiancheng Co. Ltd., Nanjing, China) and centrifuged at 13,000 g for 10 min at 4 °C. Prepare a Master Mix of the reagent according to the instructions, then add the reaction mix to each well containing the controls and samples. Mix and incubate at 25°C for hours. Add stop mix to all wells and incubate at room temperature and TNB for 5-10 min. At last, measure output (OD412 nm) on a microplate reader.

### HE Staining and Histopathological Evaluation

About 2 cm in length, the colonic tissue was excised on the ninth day after drinking DSS-water, washed in phosphate-buffered saline (PBS), embedded in 4% formaldehyde in paraffin sectioned about 2 μm, and stained with hematoxylin and eosin (H&E) (17).

### Quantitative RT-PCR

RNA was isolated from the whole colonic tissues using the Qiagen RNeasy Mini Kit following the manufacturer’s instructions. Quantitative polymerase chain reaction (qPCR) was performed for expressions of mRNAs using the primers for h*GPR174*, m*Il-6*, m*Il-10*, m*Tnf-α*, m*Occludin*, m*Zo-1*, and *β-actin*.

### Bone Marrow-Derived Dendritic Cells (BMDCs)

Bone marrow cells were extracted from femurs and tibiae of C57BL/6(n=5) and *Gpr174^-/-^* mice(n=5). After incubated in Red blood cell (RBC) lysis buffer at 37°C for 5 min, cells were seeded in six-well plates at 10^6^ cells/well concentration with RPMI-1640 medium supplemented with 20 ng/mL of granulocyte-macrophage colony-stimulating factor, 10% fetal bovine serum, 100 U/mL penicillin, and 100 mg/mL streptomycin. On day 3, the culture medium was changed to remove the nonadherent granulocyes without dislodging the cluster of dendritic cells and macrophages. On day 5, the suspension and loosely adherent dendritic cells were harvested by gently swirling the dishes to get rid of the bone-marrow derived macrophages which is tightly adherent to the dishes (18).

### Flow Cytometry

An anti-FcR antibody was used to block the non-specific staining. Cells were subsequently immunostained with APC-conjugated anti-CD11c, APC-cy7-conjugated anti-CD86, and FITC-conjugated anti-MHC-II antibodies (Biolegend, USA).

### BMDCs and Naïve CD4^+^ Cell *In Vitro* Co-Culture Assays

BMDCs were prepared as previously described and purified by CD11c^+^ magnetic beads. BMDCs were co-cultured with isolated OT-II naïve CD4^+^ T cells at a 1:10 ratio in complete RPMI in 96-well V-bottom plates. Cells were harvested 3 days later for further analysis. OVA peptide 323-339 (GenScript, USA) was added to wells at 1 mg/mL as a positive control.

### CFSE Proliferation Assay

Isolate naïve CD4^+^ T cells from OT-II mice(n=5), label with carboxyfluorescein N-succinimidyl ester (CFSE, C34554; Life Technologies), and culture in the presence of WT BMDCs and *Gpr174^-/-^* DCs. Unstimulated T cells serve as a negative control. The percentage of divided cells was analyzed by FlowJo V10.

### RNA Sequencing and Functional Analysis

Treated with LPS for 12 h or left untreated, BMDCs were harvested for RNA extraction by QIAGEN RNeasy Mini Kit. The Agilent 4200 TapeStation System was used to check RNA integrity to ensure RNA integrity was ≥ 8.9. The TruSeq Stranded mRNA Library Prep Kit (Illumina) made libraries for RNASeq using 1-1.5 μg of RNA as input. The HiSeq 4000 Sequencing Systems (Illumina) was used to run across two lanes to yield 2 × 151 bp paired-end reads with an average yield of ~55 million reads/sample. a

Fisher’s exact test was used to calculate differential gene expression. If it met the cut-off criteria, it was considered significantly differentially. An R Bioconductor package heatmap3 was used to visualize the differential gene expression (https://cran.r-project.org/package=heatmap3). DAVID (version 6.8) was used for gene term enrichment.

### Patient Enrollment

The Research and Ethics committee of Zhongshan Hospital, Fudan University, approved this study (NO: B2020-016R). All procedures were directed under guidelines. Samples from ten patients with ulcerative colitis (UC) collected under colonoscopy were used for qPCR and immunofluorescence.

### Statistical Analysis

Data were expressed as mean ± standard error (SEM). One-way ANOVA, t-test followed by Bonferroni tests was performed using GraphPad Prism 7.0. Transcriptome analysis was visualized using the R prcomp function and ggplot package. A *P* value < 0.05 was considered statistically significant.

## Results

### *Gpr174^-/-^* Mice Were Resistant to DSS-Induced Colitis

**Figure 1:**
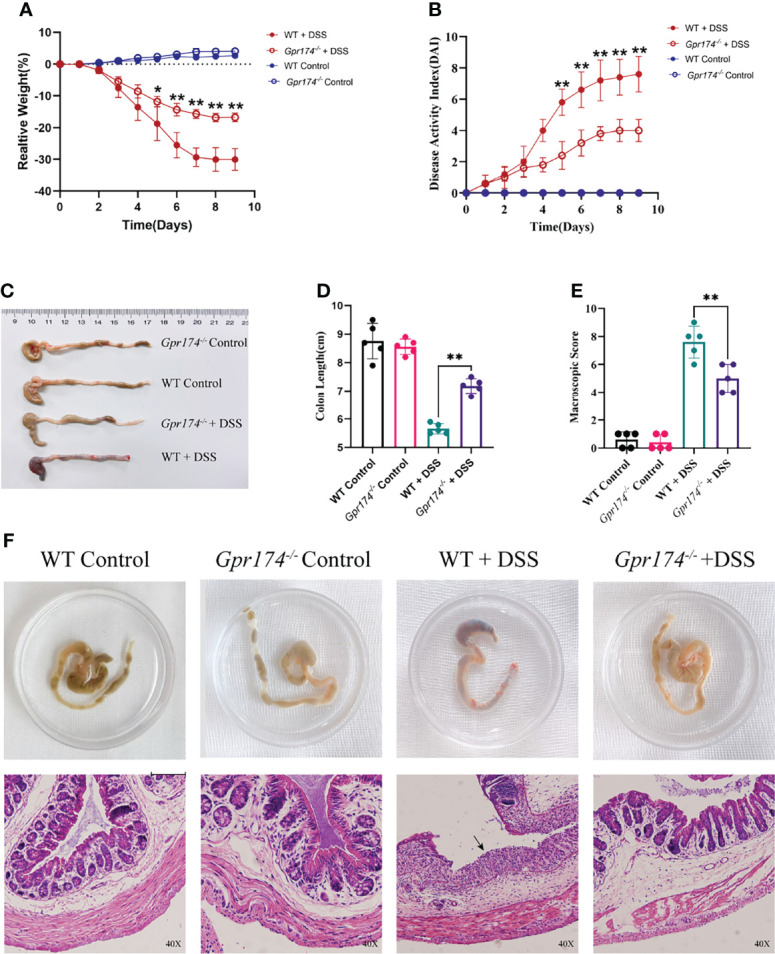
*Gpr174^-/-^* mice were resistant to DSS-induced colitis. **(A)** Body weight loss in each group, **(B)** DAI scores in each group, **(C, D)** Colon length of each group, **(E)** Macroscopic score of each group, **(F)** Morphology and HE of the intestine of each group. Data were exhibited as mean ± SEM of 5 mice per group. (Black arrow: epithelial barrier disruption. *P < 0.05, **P < 0.01).

To explore whether the GPR174 receptor is involved in the pathophysiology of IBD, we established a DSS-induced colitis animal model as previously described. After oral administration of DSS for 7 days and 2 days on regular drinking water, *Gpr174 ^-/-^* mice showed improved acute colitis symptoms compared with WT mice. *Gpr174^-/-^* mice significantly decreased DAI scores (**Figure 1B**, *P < 0.01*), with less weight loss (**Figure 1A**, *P < 0.05*) and later appearance of diarrhea loose feces. Decreased loose bloody stools and a lower reduction in colon length were shown in *Gpr174^-/-^* mice compared with the WT mice **(** **Figures 1C, D**, *P < 0.01***)**. Furthermore, compared with the WT mice, *Gpr174^-/-^* mice exhibited minor architecture damage or epithelial barrier disruption, leading to the improved macroscopic scores with DSS treatment **(** **Figures 1E, F**, *P < 0.01***)**.

### Knockout of *Gpr174* Reduced Gut Inflammatory Response and Maintained Intestinal Barrier in DSS-Induced Colitis

**Figure 2:**
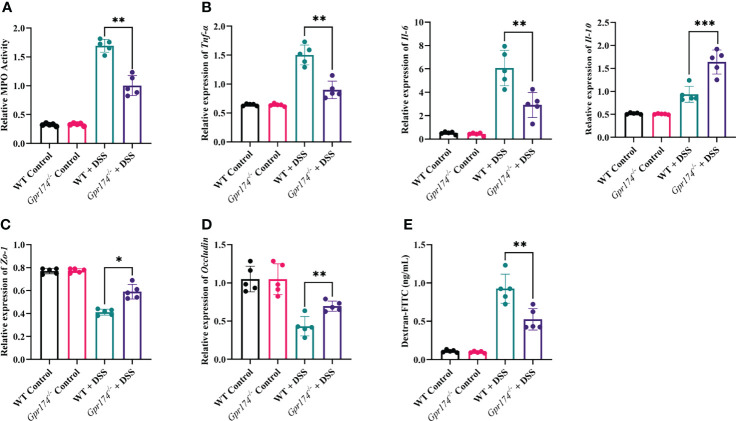
*Gpr174* knockout reduced intestinal inflammation and protected the intestinal barrier. **(A)** Intestinal MPO expression in each group, **(B)** Intestinal cytokine expression of TNF-α, IL-6, IL-10 in each group, **(C, D)** Intestinal tight junctions of *Zo-1* and *Occludin* mRNA expression in each group, **(E)** Intestinal permeability in each group. Data were exhibited as mean ± SEM of 5 mice per group. (*P < 0.05, **P < 0.01, ***P < 0.001).

The intestinal barrier damage is caused by inflammatory responses and is characterized by epithelial barrier dysfunction and increased mucosal permeability. Myeloperoxidase (MPO), reflecting the degree of neutrophil infiltration, was decreased in the distal colonic tissue of *Gpr174*^-/-^ mice compared with WT mice **(** **Figure 2A**, *P < 0.01***)**. To analyze why the *Gpr174^-/-^* mice reduced susceptibility to DSS, we evaluated the production of pro-inflammatory and anti-inflammatory cytokines in the colonic tissue. The results showed that *Gpr174^-/-^* mice treated with DSS demonstrated decreased levels of TNF-α (*P < 0.01*), IL-6 (*P < 0.01*), and increased levels of IL-10 (*P < 0.001*) compared with WT mice (**Figure 2B**). In addition, our study indicated that the expression of *Zo-1* mRNA **(** **Figure 2C**, *P < 0.05*) and *Occludin* (**Figure 2D**, *P < 0.01*) *in Gpr174^-/-^* mice was significantly higher than that in WT mice. Besides, we observed that serum FITC-dextran was markedly decreased in *Gpr174^-/-^* mice compared with WT ones 6 h after gavaging with FITC-dextran (**Figure 2E**, *P < 0.01*), suggesting that epithelial permeability was reduced in the *Gpr174^-/-^* mice.

### *Gpr174* Knockout Influenced CD11c^+^ Dendritic Cells Infiltration in Intestinal Lamina Propria(LP) in DSS-Induced Colitis

**Figure 3:**
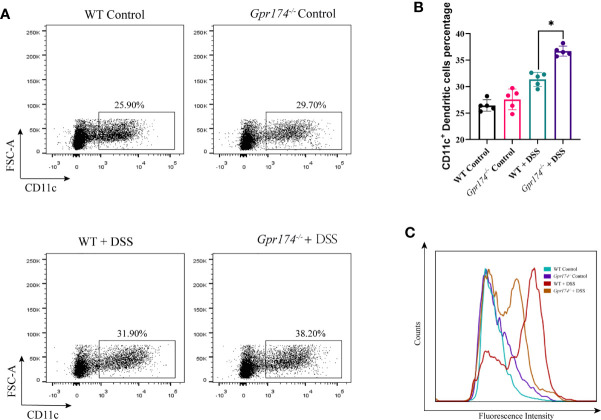
*Gpr174* knockout increased CD11c^+^ dendritic cells infiltration while suppressed maturation in the lamina propria. **(A, B)** Infiltration of CD11c^+^ dendritic cells in the lamina propria in each group, **(C)** MHC-II expression of CD11c^+^ dendritic cells in each group. Data were exhibited as mean ± SEM of 5 mice per group. (*P < 0.05).

Due to intestinal inflammation, both WT and *Gpr174^-/-^* mice exhibited increased CD11c^+^ DCs accumulation in LP compared with the WT ones after model establishment. The number of DCs in LP showed no difference between WT and *Gpr174^-/-^* mice, while the percentage of CD11c^+^ DC, one of the most potent antigen-presenting cells, increased in *Gpr174^-/-^* mice than in WT mice (**Figures 3A, B**, *P < 0.05*). However, the expression of the MHC-II molecule decreased (**Figure 3C**).

### *Gpr174* Knockout Led to Difficulty of BMDC Maturation

**Figure 4:**
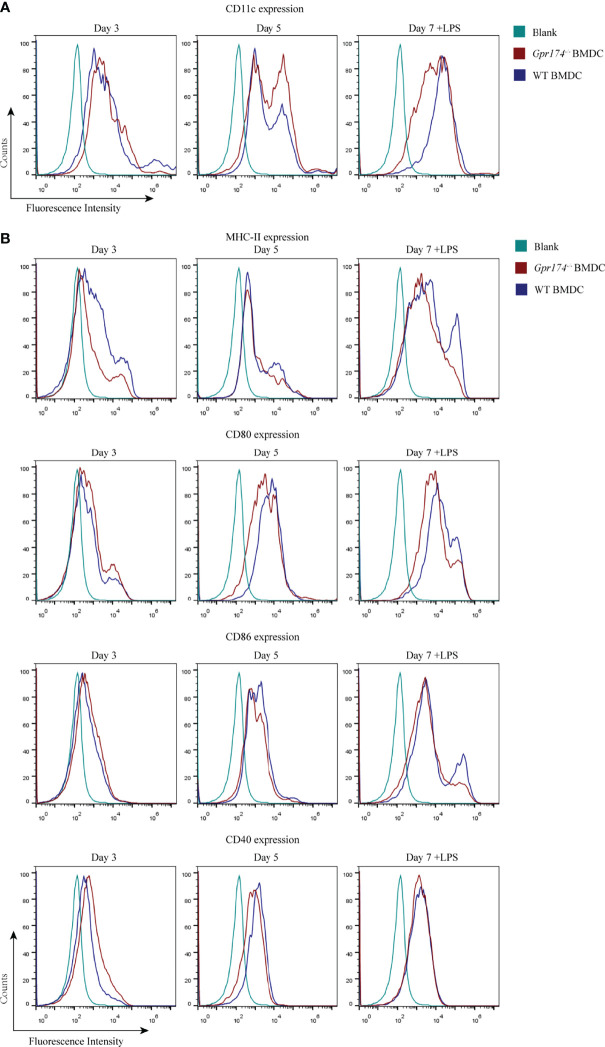
*Gpr174* knockout inhibited maturation of BMDCs. **(A)** CD11c^+^ expression of BMDCs in each group, **(B)** Co-stimulatory molecular MHC-II, CD80, CD86, CD40 BMDCs. Data were exhibited as mean ± SEM of 5 mice per group.

To further analyze the role of *Gpr174* on the immune function of CD11c^+^ DC, we examined the expression of co-stimulatory molecules of BMDCs. BMDCs extracted from the bone marrow of *Gpr174^-/-^* mice were less prone to differentiate into CD11c^+^ cells, especially on the seventh day of culture **(** **Figure 4A****)**. Both *Gpr174^+/+^* and *Gpr174^-/-^* BMDCs upregulated the expression of MHC II, CD80, CD86, and CD40 with LPS stimulation. However, the expression of MHC II, CD80, and CD86 increased much less in *Gpr174^-/-^* BMDCs than that in *Gpr174^+/+^* (**Figure 4B**).

### *Gpr174* Knockout Suppressed BMDCs to Naïve T Cell Proliferation and Differentiation

**Figure 5:**
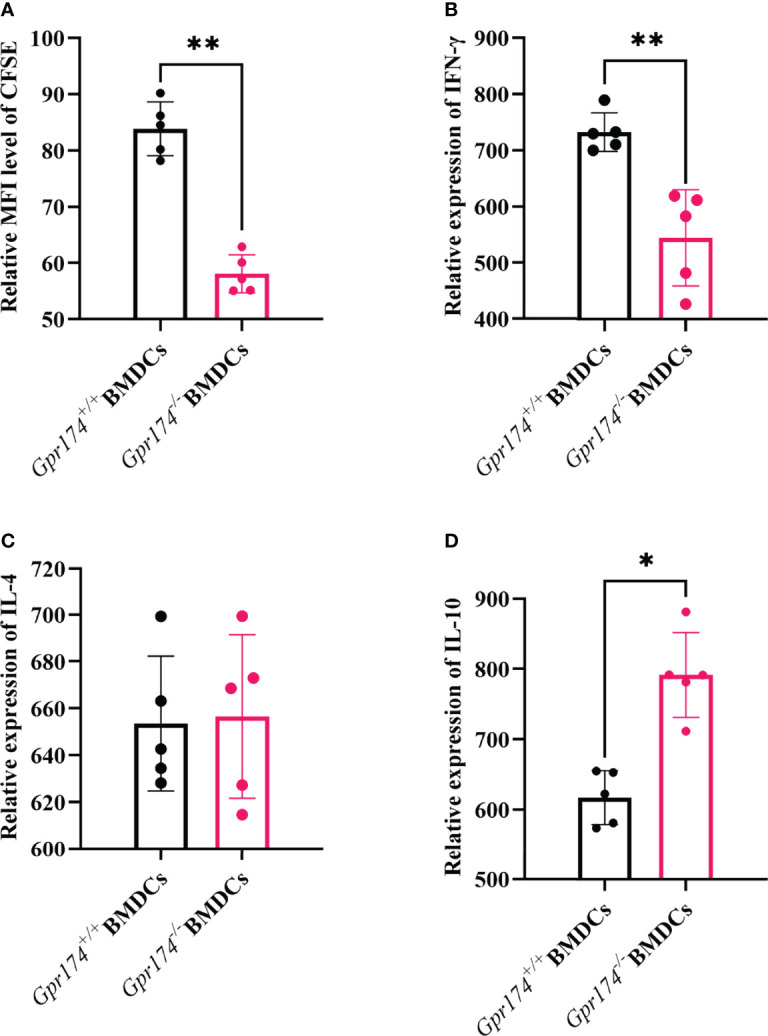
*Gpr174* knockout inhibited T cell proliferation and influenced T cell differentiation. **(A)** Degree of naive T cells proliferation, which was labeled as CFSE after stimulation in each group, **(B)** IFN-γ expression of naive CD4^+^ T cells after stimulation in each group, **(C)** IL-4 expression of naive CD4^+^ T cells after stimulated in each group, **(D)** IL-10 expression of naive CD4^+^ T cells after stimulated in each group. Data were exhibited as mean ± SEM of 5 mice per group. (*P < 0.05, **P < 0.01).

The naïve CD4^+^ T cells purified from the spleens of OT-II OVA-specific T cell receptor transgenic mice were used to examine the effects of *Gpr174* on BMDCs to stimulate naïve T cell cytokine production. *Gpr174^-/^*^-^ BMDCs induced less naïve T cell proliferation than *Gpr174^+/+^* BMDCs **(** **Figure 5A**, *P < 0.01***)**. Co-culturing with *Gpr174^-/^*^-^ BMDCs led to a significant reduction of IFN-γ (**Figure 5B**, *P < 0.01*) and an increase in the production of IL-10 in CD4^+^ T cells **(** **Figure 5D**, *P < 0.05***)**, while without any change in IL-4 **(** **Figure 5C****)**, indicating that *Gpr174^-/^*^-^ BMDCs induced more naïve CD4^+^ T cells to differentiate into Treg cells, while less into Th1 cells.

### Adoptively Transfer of *Gpr174^-/-^* BMDCs Alleviated DSS-Induced Colitis

**Figure 6:**
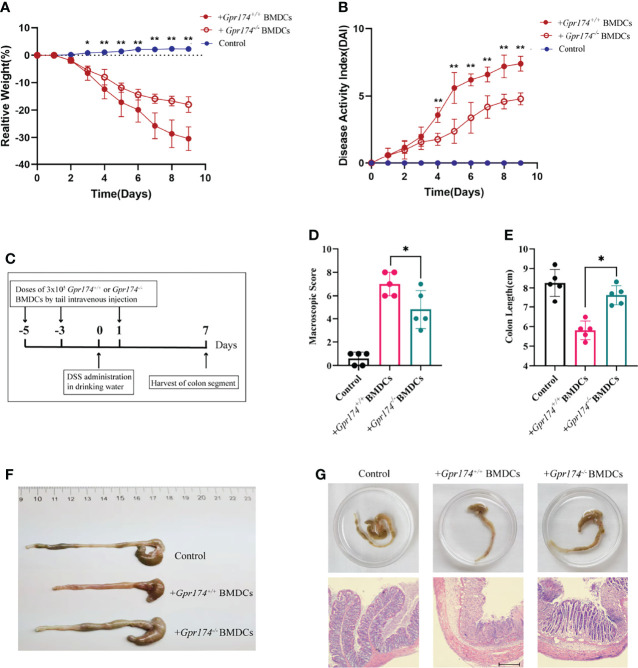
Adoptive transfer of *Gpr174^-/-^* BMDCs alleviated DSS-induced colitis. **(A)** Body weight loss in each group after adoptive transfer of *Gpr174^-/-^* DC, **(B)** DAI scores in each group, **(C)** Flow chart of the procedure of adoptive transfer of BMDCs and establishing DSS-induced colitis animal models, **(D)** Macroscopic score of each group, **(E, F)** Colon length of each group, **(G)** Morphology and HE of the intestine of each group. Data were exhibited as mean ± SEM of 5 mice per group. (*P < 0.05, **P < 0.01).

**Figure 7:**
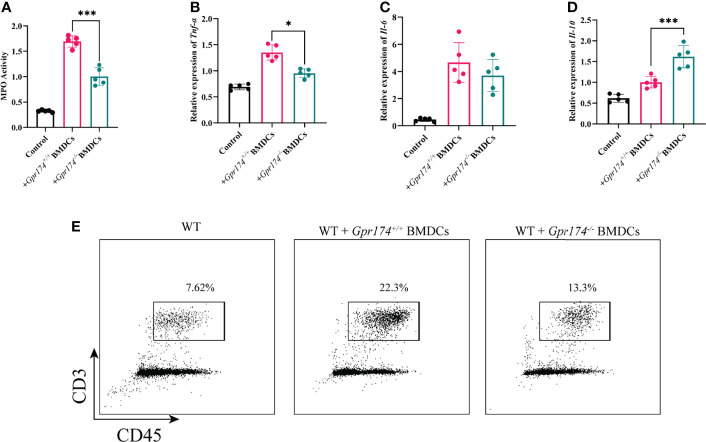
Adoptive transfer of *Gpr174^-/-^* BMDCs alleviated inflammatory response of colitis. **(A)** Colonic MPO expression in each group, **(B)** Colonic cytokine expression of TNF-α in each group, **(C)** Intestinal cytokine expression of IL-6 in each group, **(D)** Intestinal cytokine expression of IL-10 in each group, **(E)** CD3^+^ T cells infiltration in the lamina propria in each group (*P < 0.05, ***P < 0.001).

To verify the function of *Gpr174^-/-^* BMDCs in alleviating DSS-induced colitis, we adoptively transfer *Gpr174^-/-^* BMDCs and *Gpr174^+/+^* BMDCs into C57/BL6 mice intravenously at 5 days and 3 days before and 1 day after the induction of colitis, flow chart of procedure were shown in **Figure 6C**. Mice transferred with *Gpr174^-/-^* BMDCs were in a better condition after administration of DSS, characterized by the decreased weight loss **(** **Figure 6A**, *P<0.05***)**, ameliorative DAI scores **(** **Figure 6B**, *P<0.01***)**, better macroscopic scores **(** **Figure 6D**, *P<0.01***),** and reduced shortening of colon length **(** **Figures 6E, F** *P<0.05***)**. Less disruption of the epithelium in mice treated with *Gpr174^-/-^* BMDCs than the *Gpr174^+/+^* BMDCs was exhibited by H&E staining **(** **Figure 6G****)**. Besides, the inflammatory response was mitigated in the ones transferred with *Gpr174^-/-^* BMDCs, with MPO (**Figure 7A**, *P < 0.05***)**, TNF-α (**Figure 7B**, *P < 0.05***),** and IL-6 (**Figure 7C****)** declined, IL-10 (**Figure 7D**, *P < 0.001***)** increased in the colon. In addition, T cells were reduced in the LP transferred with *Gpr174^-/-^* BMDCs (**Figure 7E**, *P < 0.01*)

### *Gpr174* Knockout Altered Dendritic Cells Transcriptome After Maturation

**Figure 8:**
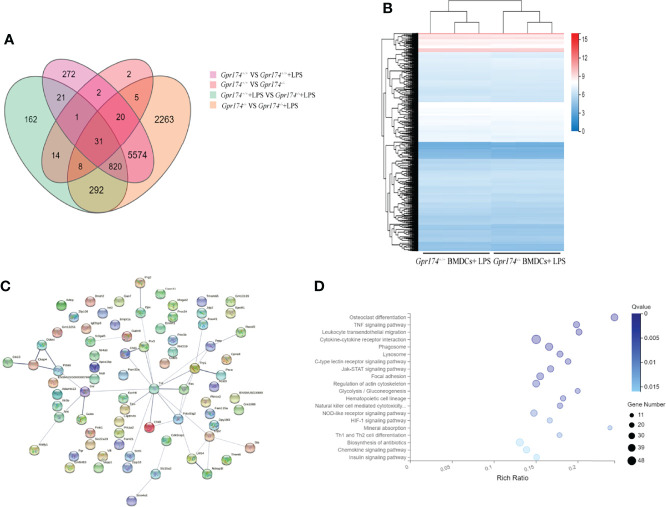
*Gpr174* knockout influences BMDCs transcriptome after LPS stimulation. **(A)** Venn diagram of differential expression genes (DEGs) of four groups of BMDCs before and after LPS application, **(B)** Heatmap of DEGs of BMDCs in each group after LPS application, **(C)** Protein-protein interactions (PPI) networks analysis of DEGs of *Gpr174^-/-^* BMDCs and *Gpr174^+/+^* BMDCs after LPS application, **(D)** KEGG analysis of DEGs of *Gpr174^-/-^* BMDCs and *Gpr174^+/+^* BMDCs after LPS application (the first twenty 20 pathways with ease < 0.05, count ≥ 10, and FDR < 0.01).

*Gpr174^+/+^* BMDCs and *Gpr174^-/-^* BMDCs were subjected to transcriptome analysis to reveal the role of *Gpr174* in the differentiation and maturation of BMDCs. LPS led to significant modulation of gene expression between *Gpr174^-/-^* BMDCs and *Gpr174^+/+^* BMDCs. However, no significant differences were observed between *Gpr174^-/-^* BMDCs and *Gpr174^+/+^* BMDCs without LPS stimulation (**Figures 8A, B****)**. TNF-α was extracted from protein to protein interaction (PPI) network analysis of DEGs to be a hub gene (**Figure 8C**). The enrichment of KEGG analysis suggested that the TNF-α (NF-κB) signaling pathway and leukocyte transendothelial migration pathway may be the potential target pathway of *Gpr174*** (** **Figure 8D****).**

### *GPR174* Expression Was Decreased in the Inflamed Mucosa of UC Patients

**Figure 9:**
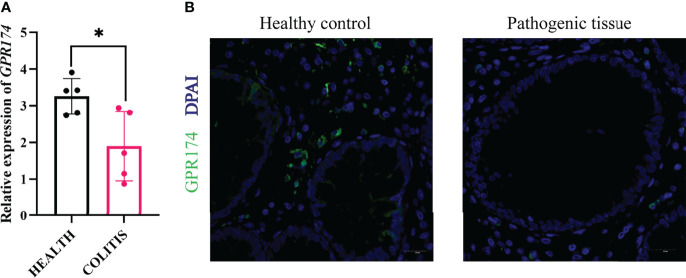
Expression of *GPR174* between healthy and pathogenic tissues. **(A)** The mRNA expression of *GPR174* between healthy and pathogenic tissues from the ulcerative patients(n=10), **(B)** The protein expression of GPR174 receptor between healthy and pathogenic tissues from the ulcerative patients (Blue: DAPI; Green: GPR174). (*P < 0.05).

To further confirm the role of *GPR174* in the pathogenesis of UC, the inflamed mucosas of the UC patients(n=10) were collected. The expression of *GPR174* mRNA was relatively lower in the pathological site than in the healthy control **(** **Figure 9A**, *P < 0.05***).** Likewise, the immunofluorescence showed less *GPR174* expression in the inflamed tissues **(** **Figure 9B****)**.

## Discussion

Our study showed that *Gpr174* knockout reduced inflammatory response and altered the phenotype of DCs in colitis intestines. *Gpr174* knockout lowered the expression of co-stimulatory molecules and influenced the ability of BDMCs to mature, which further altered the proliferation and differentiation of naïve T cells. T cell activation is a critical step in the intestinal immune system, and only the activated T cells exert an efficient mucosa immune response. Moreover, we adoptively transferred *Gpr174^-/-^* BMDCs to alleviate DSS-induced colitis and gut injury. The transcriptomic analysis revealed NF-κB signaling pathway participated in the process related to BMDCs maturation. Furthermore, the expression of *GPR174* mRNA was decreased in the inflamed mucosa in IBD patients.

GPCRs are critical signaling molecules in immune response, inflammation regulation, cell proliferation (19), cell migration (20), and intestinal barrier maintenance (8, 21). Researchers have revealed that GPR43 could bind with short-chain fatty acids (SCFAs) to regulate the inflammatory responses in intestinal diseases, which indicated that GPCRs took a critical part in the pathogenesis of IBD. Our previous study demonstrated that *Gpr174* knockout elevated the number of marginal zone B cells in the spleen of mice and promoted the function of regulatory T cells, cytotoxic T lymphocytes, and M2 macrophage polarization (11, 22). In this study, we found that deletion of *Gpr174* could generally alleviate intestinal injury in the DSS-induced colitis, which considered *Gpr174* participated in the pathogenesis of IBD.

Moreover, GPCRs also act as a navigator in the migration of dendritic cells from peripheral to draining Lymph nodes (LNs) and their maturation process (23). GPCRs such as chemokine receptors CCR7 and CCR4 are highly upregulated on DCs upon maturation. Moreover, GPCRs like GPR183 and CXCR5 help direct DCs to certain areas upon inflammation (24). DCs, a bridge between innate and acquired immune systems, maintain the homeostasis of intestinal immunity in the LP, were found to decrease colitis after *Gpr174* knockout in this study.

We chose BMDCs to investigate the role of GPR174 in regulating the function of DCs because they could be abundantly generated *in vitro* and were widely applied as a model myeloid DC (25). In our study, *Gpr174* modulated murine BMDCs maturation and migration. We found that deletion of the *Gpr174* in BMDCs hardly affected the expression of CD86, CD80, or MHC-II on non-stimulated BMDCs. However, after being stimulated by LPS, *Gpr174^-/-^* BMDCs showed a different expression profile of co-stimulatory molecules, such as CD80, CD86, and MHC-II, which were significantly decreased, indicating that *Gpr174^-/-^* BMDCs remained an immature phenotype. Immature DCs possess a solid ability to phagocytose and digest antigens and then process and present antigens and gain a mature phenotype. Once BMDCs acquire mature phenotypes, they are empowered to activate naïve T/B cells (26, 27). Mature DCs migrate from LP to lymph nodes. They initiate naïve T/B cells and induce them to proliferate and differentiate. Many studies indicated that tolerant DCs could reduce the severity of DSS-induced colitis, probably due to their cytokine profiles and weakened immunity. Activated DCs also secrete pro-inflammatory cytokines to intercede the inflammatory regulation in UC by activating TLRs, which induce infiltration of neutrophils and activation of other innate immune cells (28). Like DCs and macrophages, cytokines released by APCs and macrophages trigger naïve T/B cell differentiation into various subsets. Consistent with the alteration of maturity, *Gpr174 ^-/-^* BMDCs exhibited the impaired ability to stimulate naïve T cells to proliferation and differentiation.

Preliminary studies demonstrated that stimulation of OT-II CD4^+^ T cells co-cultured with DCs and 50 nM OVA (329-337 peptides) induced a high expression level of both IFN-γ and IL-4 a modest level of IL-10 (29, 30). *In vitro* experiments, our study indicated that *Gpr174^-/-^* BMDCs were less capable of stimulating naïve CD4^+^ T cells. Lumen antigens were endocytosed, digested by DCs, and then presented to naïve T/B cells to initiate systemic and mucosal immunity (31). Activated DCs in LP would acquire maturity and migrate to mesenteric lymph node (MLN) and secondary lymphoid tissues with antigens to translate innate to adaptive immune response. In addition, effector T cell subsets which were differentiated after binding with DCs also changed. Following activation, naïve CD4^+^ T cells differentiated into several types of effector T cells, subdivided into either Th1, Th2, or Treg cells according to cytokine profiles.

The pathogenesis of IBD may be an excessive activation of effector T cells and alteration of T cell-mediated tolerance, which is related to Treg development or alteration (32). An animal experiment showed that SAMP1/YitFc mice, which had massively expanded B cells in the intestine, developed transmural gut inflammation by blocking Tregs’ immune function (33). Helper T cells are critical mediators of the immune response, while regulatory T cells have a robust immune suppression function. Recent studies demonstrated that the immune imbalance between CD4^+^ T cells, Th1/Th2, and Treg might be the most direct and vital factor in the pathogenesis of IBD. It also played a role in mediating the occurrence of ulcerative colitis (34, 35). The current study suggested a reducing ratio of Th1/Th2 and Treg in LP alleviated inflammatory response in colonic tissue. Less effector T cells reduced pro-inflammatory factors release, thereby alleviating intestinal damage.

Pro-inflammatory cytokines released in the gut are involved in IBD and have different functions, including cellular adhesion, differentiation, and transmigration (36). Our results strongly suggested that increased pro-inflammatory cytokines in the gut of mice treated with DSS accounted for the observed exacerbation of colitis. Our study showed that *Gpr174*^-/-^ mice with DSS treatment suppressed the pro-inflammatory factors like TNF-α and IL-6 and promoted the anti-inflammatory factor IL-10. A delicate balance of pro-and anti-inflammatory factors is necessary to preserve intestinal homeostasis. The increase of IL-10 in the *Gpr174*^-/-^ mice might provide compensative mechanisms for improvements in inflammation status.

Cytokines could disrupt the intestinal epithelium, promote barrier permeability, and modulate the tight junctions (TJs). TJs are important for barrier integrity and play an essential role in intestinal homeostasis. It is reported that TNF-α on the intestinal epithelium could disrupt the epithelial barrier and decrease the tight junction proteins expression, including occludin and Zo-1 (37, 38). Once immune cells entered intestinal mucosa through broken TJs, the raised TNF-α and IFN-γ triggered cytokine storm made the intestinal barrier disruption heavier (39–41). This study showed increased occludin and Zo-1 mRNA levels and reduced macromolecule permeability in *Gpr174^-/-^* intestinal mucosa compared with *Gpr174* ^+/+^ ones, indicating *Gpr174* knockout helped maintain the intensity of the intestinal mucosa in DSS treated mice.

To further validate whether *Gpr174^-/-^* BMDCs have protective effects on IBD, we transferred *Gpr174^-/-^* BMDCs into DSS-induced colitis animals. Adoptive transfer of *Gpr174^-/-^* BMDCs could significantly improve intestinal damage and suppress the inflammatory response in both innate and acquired immunity. Some clinical research revealed that patients with active IBD lacked immature peripheral blood plasmacytoid and myeloid DCs (42). DCs with immature phenotype could induce immune tolerance by inducing T regulatory cells and amplifying suppressor activity, which may have anti-inflammatory effects (43).

The progression of IBD is closely related to the immune system (44). Animal studies showed that the *GPR174* was involved in IBD’s pathogenic course; some clinical evidence must be unearthed. Therefore, we took samples from ulcerative patients and set the non-lesion site as healthy controls to combine with clinical practice. The mRNA expression of *GPR174* decreased in the inflamed tissues compared with healthy controls, which was consistent with the immunofluorescence results. This might be because the samples we collected were the patients in the late disease courses of colitis. We stimulated BMDCs of WT mice with LPS to validate this idea and found *Gpr174* increased in the first 24h. Moreover, we collected the healthy controls’ PBMC to induce the monocyte-derived DCs (MODCs) and found the GPR174 began to increase in the 12h after LPS stimulation *in vitro*. Therefore, in the early course of LPS stimulation and colitis, the *GPR174* might increase, while in the late course of the disease, the *GPR174* expression decreased (**Figure S1**). In-depth studies are required to confirm these observations further.

Furthermore, we did some transcriptome analysis of *Gpr174^+/+^* BMDCs and *Gpr174^-/-^* BMDCs, which enhanced the speculation that *Gpr174* mainly participated in the complete maturation process. Studies showed that TNF-α cytokines and the NF-κB signaling pathway were indispensable elements in the differentiation and maturation process in BMDCs (45, 46). In this study, we found that the expression of TLR4, MYD88, IKKα, IκB-α, and p-NF-κB were decreased in *Gpr174*^-/-^ with LPS stimulation. Taken together, we considered that deletion of *Gpr174* might inhibit the NF-κB pathway and suppress BMDCs maturation.

In summary, our work revealed that the knockout of GPR174 could reduce intestinal inflammation and repair the epithelium barrier by suppressing DCs maturation and naïve T cell differentiation to alleviate DSS-induced colitis, which was probably through inhibiting the TNF-α (NF-κB) pathway.

## Data Availability Statement

The datasets presented in this study can be found in online repositories. The names of the repository/repositories and accession number(s) can be found below: https://www.ncbi.nlm.nih.gov/bioproject/, PRJNA819571.

## Ethics Statement

The studies involving human participants were reviewed and approved by NO:B2020-016R. The patients/participants provided their written informed consent to participate in this study. The animal study was reviewed and approved by NO. 201804001Z.

## Author Contributions

ZS and WW designed the research plan. WW, SM and YH conducted the experiments, analyzed all data, and wrote the manuscript. WW, SM and YH were co-first authors. ZK helped with the mice experiment. YC and YL assisted with some data analysis. YZ and XW collaborated to collect endoscopy biopsies. CT and YY supervised the experimental work and data analysis. ZS, YY and YZ were co-corresponding authors. All authors participated in revising the manuscript and agreed to the final version.

## Funding

This work was supported by the National Natural Science Foundation of China (Grant No. 82072214), National Key Research and Development Program of China (2021YFC2501800), Science and Technology of Shanghai Committee (Grant No. 20Y11900100, Grant No. 21MC1930400, Grant No.20DZ2261200), and Shanghai Municipal Health Bureau (Grant No. ZXYXZ-201906, Grant No. GWV-10.2-XD04).

## Conflict of Interest Statement

The authors declare that the research was conducted in the absence of any commercial or financial relationships that could be construed as a potential conflict of interest.

## Publisher’s Note

All claims expressed in this article are solely those of the authors and do not necessarily represent those of their affiliated organizations, or those of the publisher, the editors and the reviewers. Any product that may be evaluated in this article, or claim that may be made by its manufacturer, is not guaranteed or endorsed by the publisher.

## Abbreviations

AAD, autoimmune Addison’s disease; BMDCs, bone-marrow-derived dendritic cells; DAI, disease activity index; DC, dendritic cells; GPCRs, G protein-coupled receptors; IBD, inflammatory bowel disease; SCFAs, short-chain fatty acids; TJ, tight junction.
