# Supplementary figures and images for: Gpr174 Knockout Alleviates DSS-Induced Colitis via Regulating the Immune Function of Dendritic Cells
Source: Front Immunol. 2022 May 20;13:841254. doi: 10.3389/fimmu.2022.841254 (PMC9164256; doi:10.3389/fimmu.2022.841254)

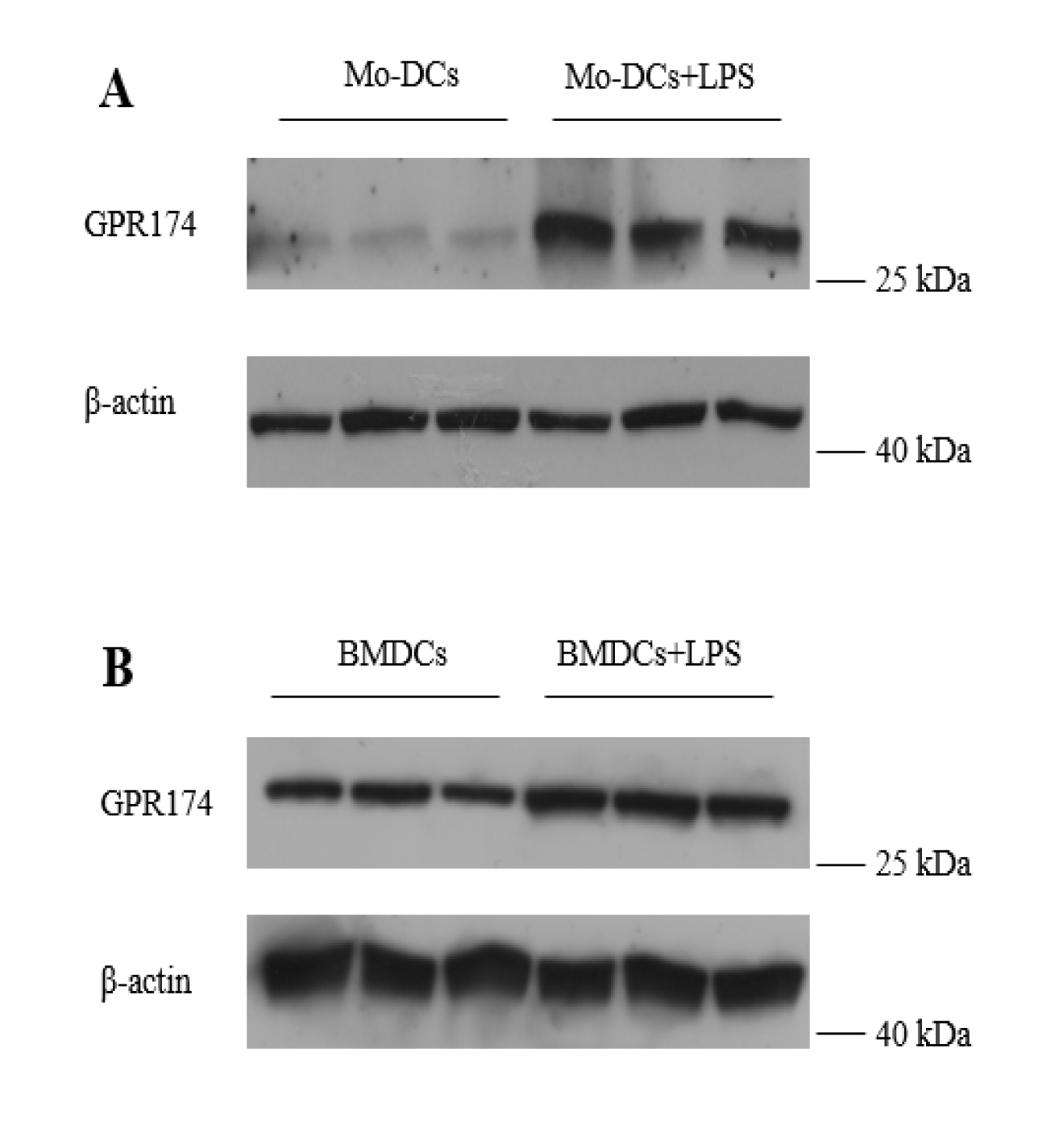

Supplement: Supplementary file 1 [file Image_1.tif]
